# Frequency of potential interactions between drugs in medical prescriptions in a city in southern Brazil

**DOI:** 10.1590/S1516-31802009000400005

**Published:** 2009-12-07

**Authors:** Genici Weyh Bleich, Ariana Bleich, Priscila Chiamulera, Andréia Cristina Conegero Sanches, Deborah Sandra Leal Guimarães Schneider, Jorge Juarez Vieira Teixeira

**Affiliations:** 1 Specialist pharmacist, Community Pharmacy, Cascavel, Paraná, Brazil.; 2 Undergraduate pharmacy student, Center for Medical and Pharmaceutical Sciences, Universidade Estadual do Oeste do Paraná (Unioeste), Cascavel, Paraná, Brazil.; 3 MSc. Pharmacist and professor, Center for Medical and Pharmaceutical Sciences, Universidade Estadual do Oeste do Paraná (Unioeste), Cascavel, Paraná, Brazil.; 4 MSc. Statistician and professor, Center for Exact and Technological Sciences, Universidade Estadual do Oeste do Paraná (Unioeste), Cascavel, Paraná, Brazil.; 5 PhD. Pharmacist and professor, Department of Clinical Analyses, Sciences Center of the Health, Universidade Estadual de Maringá (UEM), Maringá, Paraná, Brazil.

**Keywords:** Adult, Aged, Drug interactions, Pharmacoepidemiology, Prescriptions, drug, Adulto, Idoso, Interações de medicamentos, Farmacoepidemiologia, Prescrição de medicamentos

## Abstract

**CONTEXT AND OBJECTIVE:**

Drug interactions form part of current clinical practice and they affect between 3 and 5% of polypharmacy patients. The aim of this study was to identify the frequency of potential drug-drug interactions in prescriptions for adult and elderly patients.

**TYPE OF STUDY AND SETTING:**

Cross-sectional pharmacoepidemiological survey in the Parque Verde housing project, municipality of Cascavel, Paraná, Brazil, between December 2006 and February 2007.

**METHODS:**

Stratified cluster sampling, proportional to the total number of homes in the housing project, was used. The sample consisted of 95 homes and 96 male or female patients aged 19 or over, with medical prescriptions for at least two pharmaceutical drugs. Interactions were identified using DrugDigest, Medscape and Micromedex softwares.

**RESULTS:**

Most of the patients were female (69.8%), married (59.4%) and in the age group of 60 years or over (56.3%), with an income less than or equal to three minimum monthly salaries (81.3%) and less than eight years of schooling (69.8%); 90.6% of the patients were living with another person. The total number of pharmaceutical drugs was 406 (average of 4.2 medications per patient). The drugs most prescribed were antihypertensives (47.5%). The frequency of drug interactions was 66.6%. Among the 154 potential drug interactions, 4.6% were classified as major, 65.6% as moderate and 20.1% as minor.

**CONCLUSION:**

The high frequency of drug prescriptions with a potential for differentiated interactions indicates a situation that has so far been little explored, albeit a reality in household surveys.

## RESUMO

**CONTEXTO E OBJETIVO:**

As interações medicamentosas fazem parte da prática corrente na clínica médica e a sua incidência oscila entre 3% e 5% nos pacientes fazendo uso da polifarmácia. O objetivo do estudo foi identificar a frequência de interações droga-droga potenciais em prescrições de pacientes adultos e idosos.

**TIPO DE ESTUDO E LOCAL:**

Inquérito farmacoepidemiológico de corte transversal, realizado de 12/2006 a 02/2007 no conjunto habitacional Parque Verde, Cascavel, Paraná, Brasil.

**MÉTODO:**

A amostra foi por conglomerado e estratificada, proporcional ao total de residências no conjunto habitacional, constituída de 95 residências, com 96 pacientes de 19 anos ou mais, sexo feminino e masculino, com prescrição de no mínimo duas especialidades farmacêuticas. As interações foram identificadas de acordo com os softwares DrugDigest, Medscape, Micromedex.

**RESULTADOS:**

A maioria dos pacientes pertencia ao gênero feminino (69,8%), em situação conjugal casada (59,4%), com renda menor ou igual a três salários mínimos (81,3%), faixa etária 60 anos ou mais (56,3%) e 8 anos ou menos de estudo (69,8%) com 90,6% dos pacientes moravam com mais outra pessoa. O total de especialidades farmacêuticas foi de 406, correspondendo a uma média de 4,2 medicamentos por paciente. Os fármacos mais prescritos foram os anti-hipertensivos (47,5%). A frequência de interações medicamentosas foi de 66,6%. Dentre as 154 interações medicamentosas potenciais, 4,6% foram classificadas como maior e 65,6% como moderada e 20,1% como menor.

**CONCLUSÃO:**

A alta frequência de prescrições de drogas com potencial de interações diferenciado indica uma situação ainda pouco explorada, mas real em pesquisa domiciliar.

## INTRODUCTION

Simultaneous administration of several different drugs may cause significant changes in the effects brought about by their components. Interactions may increase the pharmacological effects at a toxic level, or they may inhibit the pharmacological effects and annul the patient’s therapeutic benefit.^1^ Drug interactions occur commonly in clinical practice and their frequencies range from 3 to 5% among patients presenting polypharmacy. They may even reach 20% among patients with daily use of more than 10 drugs.^2^ As a rule, drug-drug interactions are the cause of about 3.8% of hospitalizations^3^ and they may cause several adverse events in patients.^4^

Drug interactions consist of several mechanisms, which may be classified (1) as pharmacokinetic mechanisms, when one drug interferes with the absorption, distribution, metabolism or excretion of another drug, or (2) as pharmacodynamic mechanisms, when drugs with similar effects are administered together with the occurrence of either synergism or opposition of their effects that reduces the reaction to one or both drugs. A third interaction classification may occur *in vitro* when one or both drugs are inactivated.^5^^,^^6^^,^^7^

Physicians may foresee potential interactions if they understand the basic pharmacokinetic principles and the characteristics of each drug. The steps required comprise careful monitoring of patients, changes to the doses of one or both drugs, or exchanging of one or both drugs to lessen the possible interactions.^8^

Vonbach et al.^9^ reported that to reduce the number and improve the management of drug-drug interactions (DDIs), physicians primarily have to be aware of the presence of a DDI. Cruciol-Souza and Thomson^10^ reported that education for healthcare professionals, computerized systems for prescriptions and drug information, along with collaborative drug selection and pharmaceutical care are some of the possible solutions for the problem. It should be emphasized that within the therapeutic chain, the relationship between physicians and pharmacists prevents drug interactions.^11^

## OBJECTIVE

The objective of this study was to identify the frequency of potential drug-drug interactions in prescriptions for adult and elderly patients.

## METHODS

### Design and setting

A cross-sectional pharmacoepidemiological survey was undertaken in the municipality of Cascavel, Paraná, Brazil, between December 2006 and February 2007. The estimated population of this municipality is 285,784 (Instituto Brasileiro de Geografia e Estatística, IBGE, 2008),^12^ and it is divided into 31 districts. The present survey was conducted in the Parque Verde district, which comprises many housing complexes. Among these, the Parque Verde housing project, consisting of 486 houses and a population of 1,458 inhabitants, was chosen for the study. This specific area, with single-storey houses only, thus providing easy access for researchers, features a nearby primary healthcare unit (PHU) that is frequented by the inhabitants. Since this study was developed in homes and not in the PHU, all the prescribed medicines used by the population could be identified. In fact, the patients could also have prescriptions from specialist physicians attending other clinics, thus increasing the possibilities for potential drug interactions. The present study was initially assessed and approved by the Research Ethics Committee of the Universidade Estadual do Oeste do Paraná (Unioeste), under permit no. 238/2006.

### Sample characterization

**Figure 1.**
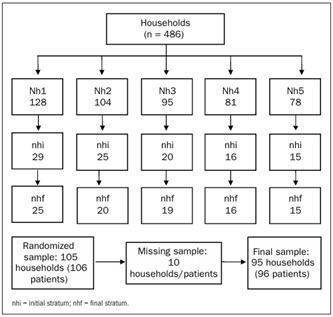
Characterization of sample of households in Parque Verde housing project, Cascavel, PR, Brazil.

A stratified cluster sample was taken, proportional to the total number of homes in the housing project. The residential complex was divided into five strata; the homes were numbered and classified with the due proportions. The randomized sample consisted of 95 homes, and this was increased to 105 in order to have a 10% safety margin for the sample. Ten homes had to be discarded due to lack of information about the patients, refusals and non-fulfillment of the inclusion criteria (at least 19 years of age and at least two prescriptions for pharmacological drugs). In spite of these exclusions, the minimum number of sample units for each stratum was achieved within the limit of the sample (Figure 1). The following formula was used to calculate the final sample size:

Nh/N = nh/n 

Where: Nh = size of population stratum; N = size of population; nh = size of sample stratum; and n = size of sample.

### Procedure

Data collection was undertaken by two undergraduate pharmacy students from the Universidade Estadual do Oeste do Paraná (Unioeste), who had previously been trained by the research coordinators. They met the patients in their homes and asked them about their interest in participating in the research. The research aims were explained to the patients, including assurances regarding secrecy and anonymity, and all participants signed a free and informed consent statement before inclusion in the study.

Information was collected using structured forms that had previously been tested in similar populations. The variables of sex, age, schooling, income, marital status, social isolation and prescribed drugs were recorded. The person interviewed in each home was someone who was actually present at the time of the researcher’s visit and who fulfilled the preestablished inclusion criteria.

### Data evaluation

Interactions were identified using the DrugDigest^®^, Medscape^®^ and Micromedex^®^ software (series 2007 and 2008).^13^^,^^14^^,^^15^ After drug interactions had been identified, two researchers (GW and JT) independently confirmed the reactions that the undergraduates had reported, in order to ensure information quality. All interactions were graded according to their severity and were confirmed by at least two types of software. Only the interactions with a high degree of severity were selected for analysis, according to their clinical importance. Vonbach et al.^9^ reported that greater sensitivity might occur when two or more investigation programs relating to drug-drug interactions are combined. The drugs were classified in accordance with the Anatomical Therapeutic Chemical (ATC) System.^16^ The data were recorded in the Epi Info 3.4.3 software, and the statistical analysis was descriptive. Data quality was ensured through rerecording and revalidating the drug interactions observed by different independent researchers.

## RESULTS

The sociodemographic characteristics of the 96 patients investigated are shown in Table 1. Most of the patients were female (69.8%), married (59.4%), aged 60 years or over (56.3%) and living with other people (90.6%), with an income of less than or equal to three minimum monthly salaries (81.3%) and less than eight years of schooling (69.8%).

Four hundred and six different drugs had been prescribed and the mean number of drugs for each patient was 4.2, with a range from 2 to 13. Table 2 shows that drugs acting on the cardiovascular system accounted for 47.5% of these drugs and were the type most prescribed by physicians.

**Figure 2.**
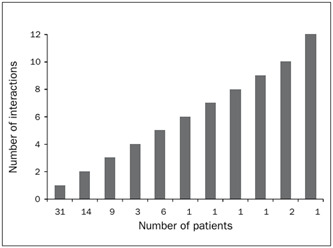
Frequency of potential drug interactions in prescriptions for adult and elderly patients.

Drug interactions were reported in the cases of 64 patients, and thus the frequency of interactions was 66.7%. Since 91 out of the total of 154 drug interactions were different, these patients may have been simultaneously subjected to between 1 and 12 interactions (Figure 2).

Taking the severity of the drug interactions into account, 7 (4.6%) were classified as major (Table 3); 101 (65.6%) as moderate; 31 (20.1%) as minor; and 15 (9.7%) were not described in the literature.

**Table 1.** Sociodemographic characteristics of the adult and elderly patients who used pharmaceutical drugs

Variables	n (96)	%	Variables	n	%
Gender			Conjugal situation		
Male	29	30.2	Married	57	59.4
Female	67	69.8	Unmarried	39	40.6
Family income^*^			Social isolation		
≤ 3	78	81.3	Living alone	9	9.4
> 3	18	18.7	Living with other people	87	90.6
Age (years)			Education (years)		
19 to 59	42	43.7	≤ 8	67	69.8
60 to 99	54	56.3	> 8	29	30.2

*In Brazilian minimum monthly salaries (US$ 165).

**Table 2.** Distribution of prescribed drugs to adult and elderly patients, according to the anatomical therapeutic chemical classification system (Anatomical Therapeutic Chemical, ATC)^16^

ATC	Medical prescription n (%)
Cardiovascular system (C)	93 (47.5)
Alimentary tract and metabolism (A)	50 (12.3)
Nervous system (N)	43 (10.6)
Musculoskeletal system (M)	36 (8.9)
Blood and blood-forming organs (B)	31 (7.6)
Respiratory system (R)	5 (1.2)
Systemic hormonal preparations (H)	17 (4.2)
Genitourinary system and sex hormones (G)	14 (3.5)
Sensory organs (S)	6 (1.5)
Anti-infectives for systemic use (J)	4 (1.0)
Various (V)	7 (1.7)
Total	406 (100)

**Table 3:** Distribution of drug-drug interactions detected using three software methods in prescriptions for adult and elderly patients, according to potential severity

Prescription drugs		n	Potential severity		
			Drugdigest Medscape Micromedex		
Propranolol +	Methyldopa	4	Major	Major	Moderate
Propranolol +	Clonidine	1	Major	Major	Major
Digoxin +	Amiodarone	1	Major	Major	Major
Warfarin +	Amiodarone	1	Major	Major	Moderate

## DISCUSSION

The mean number of prescribed drugs reported in the present study, i.e. 4.2 (standard deviation, SD = 3.0), is close to findings from other studies on patients living within the community, namely 3.2 (SD = 2.5)^17^ and 3.2 (SD = 2).^18^

Cardiovascular system drugs accounted for the largest proportion of the prescriptions (47.5%), although different results were reported in other studies undertaken elsewhere in Brazil: Fortaleza, Ceará (29.3%);^19^ Porto Alegre, Rio Grande do Sul (32.0%);^16^ and Bambuí, Minas Gerais (36%).^20^

The frequency of potential risk of developing drug-drug interactions was 66.7% and the frequency of highly severe interactions reached 4.6%, similar to the proportions reported in other studies. In an investigation on outpatients over the age of 50 years at family clinics in Mexico, Doubova Dubova et al.^21^ reported that the prevalence of potential risk was 78.8%, including a rate of high-severity interactions of 3.8%. A study at a government hospital in São Paulo, Brazil, involving patients who were prescribed antidepressive drugs showed that 21.3% were prone to develop potential drug interactions, including 5% with high-severity interactions.^8^ In a pharmacoepidemiological study, Cruciol-Souza and Thomson^10^ reported a frequency of prescription interactions of 49.7%, including 3.4% with high-severity interactions. Although different studies in different locations have reported different characteristics, the data are not contradictory, especially with regard to high-severity risk. It is important to note that the data from the present study were compared with research results in which a single type of software was used. Consequently, the present study may show higher potential risks of drug interactions.

The interaction that recurrently presented the highest-severity potential was between methyldopa and beta-adrenergic blocking agents. Methyldopa causes sedation and depression of the central nervous system.^22^ Propranolol associated with clonidine may cause a severe hypertensive crisis when clonidine is abruptly suspended.^23^^,^^24^ Although digoxin had been prescribed to only four patients, the drug had 16 entries among the interactions. One of them was seen to be of major severity. When digoxin is associated with amiodarone, significant drug interactions may occur,^25^ since it reduces renal excretion and increases plasma levels.^5^ Digoxin therapy should be adjusted based on any signs and symptoms of digoxin toxicity.^26^ Another potential interaction occurred between warfarin and amiodarone, with the potential for anticoagulant effects due to amiodarone and higher risk of hemorrhage. In fact, the warfarin dose had to be modified.^24^^,^^27^^,^^28^^,^^29^

Considering the therapeutic tools available to healthcare professionals, physicians and pharmacists in particular need to be alert regarding detection of clinically significant drug-drug interactions. Cahill^11^ reported that, because of the close links between physicians and pharmacists, teamwork for revision and intervention within the context of drug-drug interactions is required. Moura et al.^30^ stated that coordinated discussions between physicians, pharmacists and nurses is highly important for judicious evaluation of therapeutic schemes. Furthermore, the present study was the first investigation, within the household context, to use three types of software for the risk analysis on drug interactions. This practice increases the sensitivity of the software towards corroborating the results relating to drug interactions.

Among the limitations to the current investigation is the fact that it only allowed an approximation to the real issue of drug interactions within the home. Only prescribed drugs were taken into account, and there is little doubt that this will not precisely reflect the reality within these households. It is very important to underline the fact that all the visits were undertaken during normal working hours. Since this implies that there was a high likelihood of interviewing females and elderly people, it needs to be taken into account with regard to generalization of the data obtained. Further studies must be undertaken, especially in relation to drugs that are sold without prescriptions. Inclusion of these drugs may worsen the potential risk of clinically relevant interactions. The randomized sampling was limited to a single housing project and, consequently, care should be taken in generalizing these results.

## CONCLUSION

The high frequency of drug prescriptions with a potential for differentiated interactions indicates a situation that has so far been little explored, albeit a reality in household surveys.

It is highly prudent for healthcare professionals to pay more attention not only to the prescription but also to the pharmacotherapy dispensed, in order to reduce the possibility of clinical interaction and provide patients with greater benefit from treatments.
